# Is there a dose response relationship for clodronate in the treatment of tumour induced hypercalcaemia?

**DOI:** 10.1038/sj.bjc.6600249

**Published:** 2002-04-22

**Authors:** S Shah, J Hardy, E Rees, J Ling, B Gwilliam, C Davis, K Broadley, R A'Hern

**Affiliations:** Department of Palliative Medicine, The Royal Marsden NHS Trust, Sutton SM2 5PT, Surrey, UK; Department of Computing and Statistics, The Royal Marsden NHS Trust, Sutton SM2 5PT, Surrey, UK

**Keywords:** clodronate, dose response, hypercalcaemia

## Abstract

Eighty-six patients with tumour induced hypercalcaemia were randomised to 600, 900, 1200 or 1500 mg of intravenous clodronate, according to post hydration serum calcium levels. Sixty-seven were evaluable for response. The overall response rate was 49.3% (95% CI: 36.8–61.8) with no significant difference in response rates, i.e. achievement of normocalcaemia at days 6–9 (corrected serum calcium ⩽2.6 mmol l^−1^) across all groups.

*British Journal of Cancer* (2002) **86**, 1235–1237. DOI: 10.1038/sj/bjc/6600249 www.bjcancer.com

© 2002 Cancer Research UK

## 

Bisphosphonates are potent inhibitors of osteoclast activity and have become standard treatment for TIH (Body, 2000). Several studies have explored dose response relationships for different bisphosphonates and degree of hypercalcaemia (Body *et al*, 1987, 1994, 1999; Thiebaud *et al*, 1988; Nussbaum *et al*, 1993; Pecherstorfer *et al*, 1996; Ralston *et al*, 1988, 1997). There have been few studies however exploring this dose response relationship for single dose clodronate, the majority of which have involved consecutive daily clodronate infusions rather than single dose comparisons (Adami *et al*, 1987; Urwin *et al*, 1987; Bonjour *et al*, 1988; Kanis *et al*, 1990). At this tertiary cancer centre, single dose clodronate is currently used as first line treatment for TIH. The aim of this study was to evaluate its dose response relationship in TIH.

## METHODS

This was a partially, randomised, non-blinded study, comparing four doses of intravenous clodronate in patients admitted with TIH in a tertiary cancer centre. Local research and ethics committees approved the study protocol. Patients were eligible for the study if: (1) they were aged over 18 years; (2) had a non-haematological malignancy; (3) were willing and able to give written informed consent; and (4) had a corrected serum calcium level ⩾2.6 mmol l^−1^ after a minimum of 24 h rehydration with at least 2 l of intravenous normal saline. Patients excluded from the study included those with: (1) previous bisphosphonate treatment for hypercalcaemia; (2) hypercalcaemia as part of a ‘flare’ reaction to endocrine therapy; (3) a change in corticosteroid therapy in the previous 7 days; (4) intolerance of the initial volume load of rehydration; or (5) impaired renal function (serum creatinine ⩾1.5 times the upper limit of normal following rehydration). All demographic details, evidence of bone metastases and previous bisphosphonate treatment for bone pain were recorded for all patients. Calcium values were corrected for albumin using the formula, (corrected calcium=measured calcium+((40-serum albumin)×0.02)).

Patients were randomised to receive a single dose of clodronate by intravenous infusion in 500 ml normal saline over 3–4 h according to post-hydration corrected serum calcium. Patients with a corrected calcium level of: (1) 2.6–3.0 mmol l^−1^ were randomised to either 600, 900 or 1200 mg clodronate; (2) 3.0–3.4 mmol l^−1^ were randomised to 900, 1200 or 1500 mg; (3) 3.5–3.9 mmol l^−1^ were randomised to 1200 or 1500 mg; and (4) ⩾4.0 mmol l^−1^ were prescribed 1500 mg. Intravenous fluids were continued, at the discretion of the attending physician, until the patient was able to drink adequately. Serum levels of calcium, albumin, urea and electrolytes and liver function tests were recorded at days 0, 1, 3, 5, 7 and 9. All biochemical investigations were performed using standard automated techniques. Patients were continually assessed for adverse effects. Primary response was defined as achievement of normocalcaemia (corrected serum calcium ⩽2.6 mmol l^−1^) at days 6–9. Secondary endpoints recorded included the time to relapse and survival following treatment of hypercalcaemia. Patients failing to achieve normocalcaemia at days 6–9 were withdrawn from the study and given further treatment at the discretion of the physician in charge.

### Statistical methods

This study set out to test whether the effect seen in the study by Thiebaud *et al* (1988) could be replicated. Randomisation to all dose groups irrespective of initial serum calcium was not considered ethical, so the best compromise between an observational and randomised study was used. The sample size was such that its power to detect an effect of the same size as that of Thiebaud would have exceeded 80%. The Mann Whitney test for trend was used to test whether there was a trend in response rates across the four dose categories. The mean values presented are geometric means, i.e. they are back transformed means of the log transformed values.

## RESULTS

Eighty-six patients were recruited. Nineteen patients were excluded from analysis because of missing data in seven patients and death prior to completion of the study (10 patients). One patient was randomised but later excluded because of renal impairment; and one patient received chemotherapy after randomisation. A total of 67 patients were therefore evaluable for response. Patients were well matched for age, sex, tumour type, previous bisphosphonate treatment, and time of presentation from time of first development of bone metastases and hypercalcaemia (Table 1

**Table 1:**
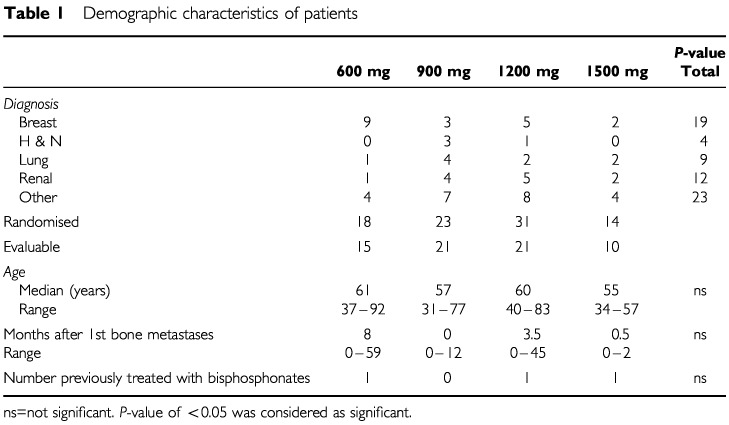
Demographic characteristics of patients

). Table 2

**Table 2:**
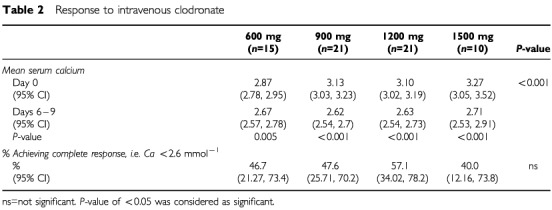
Response to intravenous clodronate

shows corrected calcium levels and response rates for the four groups. There were significant reductions in serum calcium with treatment in all four groups. There was no significant difference in response rates across the groups. The presence or absence of bone metastases (in 55% and 35% of patients respectively) had no significant bearing on response rates (*P*=0.14). There was no significant difference in response between those patients with breast (52%), lung (33%) or other malignancies (44%). Equal numbers of patients received further anti-cancer treatment with either chemotherapy, radiotherapy or hormonal treatment (mean 57%; range 46–63%). There was no difference in the median time to relapse nor median survival after treatment which was only 43 days (range 8 days to >3 years).

## DISCUSSION

In this study, there were no statistically significant difference in response rate between varying doses of clodronate. Low dose clodronate was equally effective in treating mild TIH as high dose clodronate was in treating moderate to severe TIH. Response rates were lower than that reported by O'Rourke *et al* (1993) of 80% for 1500 mg clodronate. This may be because of a relatively high proportion of patients with humoral hypercalcaemia (Kanis *et al*, 1990, 1994). Only 54% of patients in this study were known to have bone metastases and all patients with haematological malignancies (including myeloma and lymphoma) were excluded. O'Rourke *et al* (1993) found a differential response rate of only 50% in patients with humoral hypercalcaemia compared to 74% in patients with bone metastases and 100% in myeloma patients (one sixth of patients recruited). This effect has been reported by other studies (Body *et al*, 1987; Body and Dumon, 1994; Thiebaud *et al*, 1988; Ralston *et al*, 1997). In this study, no significant difference in response rate was seen between tumour types and patients with or without bone metastases but the number of patients in each group was small. Another study comparing 300 and 600 mg single infusions of clodronate reported response rates of only 17% and 40% respectively (Kanis *et al*, 1990).

In summary, this study confirms that clodronate is a safe and effective treatment for TIH but failed to confirm the dose response relationship seen in the study of Thiebaud *et al* (1988).
